# *Salmonella* Heidelberg and *Salmonella* Minnesota in Brazilian broilers: Genomic characterization of third‐generation cephalosporin and fluoroquinolone‐resistant strains

**DOI:** 10.1111/1758-2229.13132

**Published:** 2023-01-11

**Authors:** Andre Becker Simoes Saidenberg, Leticia Soares Franco, Jessica Nacarato Reple, Yaovi Mahuton Gildas Hounmanou, Monique Ribeiro Tiba Casas, Brenda Cardoso, Fernanda Esposito, Nilton Lincopan, Anders Dalsgaard, Marc Stegger, Terezinha Knöbl

**Affiliations:** ^1^ Department of Veterinary Pathology School of Veterinary Medicine and Animal Science São Paulo Brazil; ^2^ Department of Veterinary and Animal Sciences, Section for Food Safety and Zoonoses Institute for Veterinary and Companion Animal Science, Københavns Universitet Copenhagen Denmark; ^3^ Department of Bacteria, Parasites and Fungi Statens Serum Institut Copenhagen Denmark; ^4^ Bacteriology Center Adolfo Lutz Institute São Paulo Brazil; ^5^ Department of Microbiology Institute of Biomedical Science, University of São Paulo São Paulo Brazil; ^6^ Department of Clinical Analysis School of Pharmaceutical Sciences, University of São Paulo São Paulo Brazil

## Abstract

*Salmonella* serovars Heidelberg and Minnesota encoding antimicrobial resistance to third‐generation cephalosporins and fluoroquinolones are often detected in poultry/poultry meat. We analysed the genomes of 10 *Salmonella* Heidelberg (SH) and 4 *Salmonella* Minnesota (SM) from faecal isolates of Brazilian poultry. These featured virulent and multidrug‐resistant characteristics, with AmpC beta‐lactamase (*bla*_CMY‐2_) predominance (9/14), for all SM (4/4) and some SH (3/10) located on IncC plasmid replicons. IncC carrying *bla*_CTX‐M‐2_ was only detected among SH (3/10). Mutation in the *gyrA*/*parC* genes was present in all SH, whereas SM harboured *parC* mutation plus *qnrB19* on ColRNAI plasmids (3/4). In silico resistance overall corroborated with phenotypic results. Core genome phylogenies showed close clustering and high similarities between the Brazilian and poultry meat/food isolates from Europe, and to human isolates from European countries with documented import of Brazilian poultry meat. Conjugation assays with SM successfully transferred *bla*_CMY‐2_, and *qnrB19* to an *Escherichia coli* recipient. The findings reinforce the ongoing antimicrobial resistance acquisition of SH and Minnesota and the risks for disseminating resistant strains and/or mobile elements which may increasingly affect importing countries and the need for controlling AMR in major poultry‐exporting countries like Brazil.

## INTRODUCTION

Non‐typhoid *Salmonella* (NTS) remains one of the most significant zoonotic foodborne pathogens worldwide causing about 94 million cases of gastroenteritis annually and 150,000 deaths (Ao et al., 2015). NTS usually causes self‐limiting diarrheal disease with low mortality, but severe infections may be seen in immunocompromised patients and those of extremes of age, causing bacteremia, meningitis, and osteomyelitis (Tamber et al., 2021; Wen et al., 2017).

Fluoroquinolones and third‐generation cephalosporins (Wen et al., 2017) are the first‐line choice antimicrobials to treat most NTS cases. However, increased resistance to these drug classes among NTS has become a growing public health concern (GBD, 2017). As treatment options become more limited, carbapenems are being more frequently used and resistance has also been reported (Day et al., 2015).

NTS strains are often detected in poultry (Rau et al., 2021) and *Salmonella* Heidelberg (SH) Monte et al. (2019) and *Salmonella* Minnesota (SM) are frequently detected in asymptomatic birds, the farm environment, slaughterhouses, and meat products (Antunes et al., 2016; Nair et al., 2018). Both serovars are common in Brazilian poultry (Rabello et al., 2020) and have been detected in poultry products exported to the European Union (Campos et al., 2018; Liakopoulos et al., 2016; Silveira et al., 2021; van den Berg et al., 2019). Brazil is the largest exporter of poultry meat in the world (Brazilian Association of Animal Protein [ABPA], 2022) and the country may therefore play an important role in the dissemination of resistant NTS worldwide (Rabello et al., 2020).

Studies of SH and SM isolated in Brazil between 2000 and 2018 reported a variety of circulating clones from different hosts displaying genotypic resistance to multiple antimicrobials including extended spectrum beta‐lactamases (*bla*_CTX‐M_ types), AmpC beta‐lactamases (*bla*_CMY‐2_), plasmid‐mediated quinolone resistance (PMQR) genes (*qnrB*), besides chromosome mutations (*gyrA* and *parC*; Kipper et al., 2021; Monte et al., 2019; Silveira et al., 2021). In this study, we analysed the genomes of more recent isolates of the two serovars from Brazilian poultry. Virulence, and resistance features were determined, comparing the genetic relatedness of these isolates to those from other parts of the world.

## EXPERIMENTAL PROCEDURES

### Sampling

Faecal broiler samples were collected in poultry houses from different integrations (contract farms selling their products to a single company) in two Brazilian states (Sao Paulo and Minas Gerais) in the years 2019 and 2020, totalizing 400 individual samples. These were screened in aerobic cultures for *Salmonella* spp. on XLT4 agar (Oxoid, Hampshire, UK) identifying 46 strains confirmed to genus/species by MALDI‐TOF MS methodology (Bruker Daltonik, Bremen, Germany), and serotyped by macroscopic agglutination (Adolpho Lutz Institute, Brazil), according to the Kauffmann‐White‐Le Minor scheme. From this group, 10 strains identified as SH and 4 strains as SM representing different integrations were further analysed by genome sequencing.

### Minimum inhibitory concentration determination, whole‐genome sequencing, core genome phylogenies, and plasmid analyses

The methodologies for the phenotypic assays, DNA library preparation, de novo genome and plasmid assemblies, in silico typing, selection of representative genomes for comparisons, determination of the core‐genome single nucleotide polymorphisms (SNPs), and conjugation assays are described in detail in the File S1. Briefly, purified DNA was sequenced on the Illumina MiSeq Platform, and the presence of virulence and resistance markers as well as plasmid content was analysed on draft assembly genomes. Relatedness of isolates, including international isolates obtained from NCBI/EnteroBase, was performed using NASP (Sahl et al., 2016) to obtain core genome SNPs. Last, transferability of an MDR‐encoding plasmid was investigated using conjugation experiments with an *Escherichia coli* recipient.

## RESULTS

### Genotypic and phenotypic characterizations

The 14 isolates previously selected by the classical serotyping were confirmed as serovar Heidelberg (*n* = 10) and Minnesota (*n* = 4), belonging to ST15 and ST548, respectively (Table 1). For SH, isolates with a common farm origin also shared most genotypic characteristics, while the difference between farms was less pronounced in SM isolates. In silico genomic detection identified important virulence genes involved in ability of NTS to cause disease as part of *Salmonella* pathogenicity islands (SPIs), of which SPI‐1 to 14 were detected (Table 1). Detected genes included those involved in adhesion (*lpf*, *bcf*, *stb*, *stc*, *std*, and *sth*), intracellular pathogenesis as part of Type III secretion systems encoding secreted effector proteins (*inv*, *spa*, *prg*, *sip*, *spt*, *avr*, *sop*, and *slr*); Magnesium uptake (*mgtB/C*); resistance to antimicrobial peptides and survival/proliferation in the liver and spleen (*mig*‐14), oxidative stress adaptation (*sodC*); and biofilm structural formation (*fim*, *pef*, *csg*, *bcf*, *sth*, and *lpf*). Two‐component PhoPQ regulation system (*phoPQ)*, and other genes involved in the production of shock proteins (*rpo*, and *fur)* contributing to acid tolerance response were also detected in all isolates (Table 1).

**TABLE 1:** Predicted in silico characteristics of the 10 *Salmonella* Heidelberg and 4 *Salmonella* Minnesota isolates whole‐genome sequenced in this study

Isolate ID	ONE415	304	305	ONE418	297	300	ONE419	ONE420	306	307	309	318	319	320
Integration	Integration A	Integration A	Integration A	Integration B	Integration B	Integration B	Integration C	Integration C	Integration D	Integration D	Integration E	Integration F	Integration G	Integration H
Serovar	Heidelberg	Heidelberg	Heidelberg	Heidelberg	Heidelberg	Heidelberg	Heidelberg	Heidelberg	Heidelberg	Heidelberg	Minnesota	Minnesota	Minnesota	Minnesota
MLST (Achtman scheme)	15	15	15	15	15	15	15	15	15	15	548	548	548	548
SPIs	C63PI, CS54, SPI‐1, ‐2, ‐3, ‐4, ‐5, ‐9, ‐13, and ‐14	C63PI, CS54, SPI‐1, ‐2, ‐3, ‐4, ‐5, ‐9, ‐13, and ‐14	C63PI, CS54, SPI‐1, ‐2, ‐3, ‐4, ‐5, ‐9, ‐13, and ‐14	C63PI, CS54, SPI‐1, ‐2, ‐3, ‐4, ‐5, ‐9, ‐13, and ‐14	C63PI, CS54, SPI‐1, ‐2, ‐3, ‐4, ‐5, ‐9, ‐13, and ‐14	C63PI, CS54, SPI‐1, ‐2, ‐3, ‐4, ‐5, ‐9, ‐13, and ‐14	C63PI, CS54, SPI‐1, ‐2, ‐3, ‐5, ‐9, ‐13, and ‐14	C63PI, CS54, SPI‐1, ‐2, ‐3, ‐4, ‐5, ‐9, ‐13, and ‐14	C63PI, CS54, SPI‐1, ‐2, ‐3, ‐5, ‐9, ‐13, and ‐14	C63PI, CS54, SPI‐1, ‐2, ‐3, ‐4, ‐5, ‐9, ‐13, and‐14	C63PI, SPI‐1, ‐2, ‐3, ‐4, ‐5, ‐13, and ‐14	C63PI, SPI‐1, ‐2, ‐3, ‐5, ‐9, ‐13, and ‐14	C63PI, SPI‐1, ‐2, ‐3, ‐4, ‐5, ‐13, and ‐14	C63PI, SPI‐1, ‐2, ‐3, ‐4, ‐‐5, ‐13, and ‐14
Antimicrobial resistance markers	*aac(6′)‐Iaa*, gyrA* ^*1*^, parC* ^*2*^, *sul2*, *tet*(A*)*, *bla*_CMY‐2_, *fosA7*	*aac(6′)‐Iaa*, gyrA* ^*1*^ parC* ^*2*^, *sul2*, *tet*(A), *bla*_CMY‐2_, *fosA7*	*aac(6′)‐Iaa*, gyrA* ^*1*^, parC* ^*2*^, *sul2*, *tet*(A), *bla*_CMY‐2_, *fosA7*	*aac(6′)‐Iaa*, *aac(3)‐*VIa, *ant(3″)‐Ia*, gyrA* ^*1*^, parC* ^*2*^, *sul1*,*sul2*, *tet*(A), *bla*_CTX‐M‐2_, *fosA7*, *qacE*	*aac(6′)‐Iaa*, *aac(3)‐*VIa, *ant(3″)‐Ia*, gyrA* ^*1*^, parC* ^*2*^, *sul1*,*sul2*, *tet*(A), *bla*_CTX‐M‐2_, *fosA7*, *qacE*	*aac(6′)‐Iaa*, *aac(3)‐*VIa, *ant(3″)‐Ia*, gyrA* ^*1*^, parC* ^*2*^, *sul1*,*sul2*, *tet*(A), *bla*_CTX‐M‐2_, *fosA7*, *qacE*	*aac(6′)‐Iaa*, gyrA* ^*1*^, parC* ^*2*^, *sul2*, *tet*(A), *bla*_CMY‐2_, *fosA7*	*aac(6′)‐Iaa*, gyrA* ^*1*^, parC* ^*2*^, *sul2*, *tet*(A), *bla*_CMY‐2_, *fosA7*	*aac(6′)‐Iaa*, gyrA* ^*1*^, parC* ^*2*^, *sul2*, *tet*(A), *fosA7*	*aac(6′)‐Iaa*, gyrA* ^*1*^, parC* ^*2*^, *sul2*, *tet*(A), *fosA7*	*aac(6′)‐Iaa*, parC* ^*2*^, *sul2*, *tet*(A), *bla*_CMY‐2_	*aac(6′)‐Iaa*, *ant(3″)‐Ia*, *aph(3′)‐Ia*, *qnrB19*, parC* ^*2*^, *sul2*, *tet*(A), *bla*_CMY‐2_	*aac(6′)‐Iaa*, *ant(3″)‐Ia*, *aph(3′)‐Ia*, *qnrB19*, parC* ^*2*^, *sul2*, *tet*(A), *bla*_CMY‐2_	*aac(6′)‐Iaa*, *aadA22*, *ant(3″)‐Ia*, *aph(3′)‐Ia*, *qnrB19*, parC* ^*2*^, *sul2*, *tet*(A), *bla*_CMY‐2_, *formA*
Plasmids	IncX1, IncC, IncI1, and ColpVC	IncX1, IncC, IncI1, ColRNAI, and ColpVC	IncX1, IncC, IncI1, ColRNAI, and ColpVC	IncX1, IncC, and ColpVC	IncX1, IncC, ColRNAI, and ColpVC	IncX1, IncC, ColRNAI, Col156, and ColpVC	IncX1, IncC, IncI1, and ColpVC	IncX1, IncX4, IncC, IncI1, and ColpVC	IncX1, IncC, and ColpVC	IncX1, IncC, and ColpVC	IncC, ColRNAI	IncC, ColRNAI	IncC, ColRNAI	IncC, ColRNAI, IncFII
Virulence factors	*avrA*, *csg*, *fim*, *bcf*, *sth*, *lpf*, *inv*, *mgt*, *org*, *pip*, *prg*, *sif*, *sop*, *ssa*, *ssc*, *sse*, *slr*, *mig‐14*, *phoPQ*, *rpo*, *fur*, and *sodC*	*avrA*, *csg*, *fim*, *bcf*, *sth*, *lpf*, *inv*, *mgt*, *pip*, *sif*, *sop*, *ssa*, *ssc*, *sse*, *slr*, *mig‐14*, *phoPQ*, *rpo*, *fur*, and *sodC*	*avrA*, *csg*, *fim*, *bcf*, *sth*, *lpf*, *inv*, *mgt*, *pip*, *sif*, *sop*, *ssa*, *ssc*, *sse*, *slr*, *mig‐14*, *phoPQ*, *rpo*, *fur*, and *sodC*	*avrA*, *csg*, *fim*, *bcf*, *sth*, *lpf*, *inv*, *mgt*, *org*, *pip*, *prg*, sic, *sif*, *sip*, *sop*, *spa*, *ssa*, *ssc*, *sse*, *slr*, *mig‐14*, *phoPQ*, *rpo*, *fur*, and *sodC*	*avrA*, *csg*, *fim*, *bcf*, *sth*, *lpf*, *inv*, *mgt*, *pip*, sic, *sif*, *sip*, *sop*, *spa*, *ssa*, *ssc*, *sse*, *slr*, *mig‐14*, *phoPQ*, *rpo*, *fur*, and *sodC*	*avrA*, *csg*, *fim*, *bcf*, *sth*, *lpf*, *inv*, *mgt*, *pip*, sic, *sif*, *sip*, *sop*, *spa*, *ssa*, *ssc*, *sse*, *slr*, *mig‐14*, *phoPQ*, *rpo*, *fur*, and *sodC*	*avrA*, *csg*, *fim*, *bcf*, *sth*, *lpf*, *inv*, *mgt*, *org*, *pip*, *prg*, sic, *sif*, *sip*, *sop*, *spa*, *spt*, *ssa*, *ssc*, *sse*, *slr*, *mig‐14*, *phoPQ*, *rpo*, *fur*, and *sodC*	*avrA*, *csg*, *fim*, *bcf*, *sth*, *lpf*, *inv*, *mgt*, *org*, *pip*, *prg*, sic, *sif*, *sip*, *sop*, *spa*, *spt*, *ssa*, *ssc*, *sse*, *slr*, *mig‐14*, *phoPQ*, *rpo*, *fur*, and *sodC*	*avrA*, *csg*, *fim*, *bcf*, *sth*, *lpf*, *inv*, *mgt*, *org*, *pip*, *prg*, sic, *sif*, *sip*, *sop*, *spa*, *spt*, *ssa*, *ssc*, *sse*, *slr*, *mig‐14*, *phoPQ*, *rpo*, *fur*, and *sodC*	*avrA*, *csg*, *fim*, *bcf*, *sth*, *lpf*, *inv*, *mgt*, *org*, *pip*, *prg*, sic, *sif*, *sip*, *sop*, *spa*, *spt*, *ssa*, *ssc*, *sse*, *slr*, *mig‐14*, *phoPQ*, *rpo*, *fur*, and *sodC*	*avrA*, *csg*, *fim*, *pef*, *bcf*, *sth*, *lpf*, *inv*, *mgt*, *org*, *pip*, *prg*, sic, *sif*, *sip*, *sop*, *spa*, *spt*, *ssa*, *ssc*, *sse*, *slr*, *mig‐14*, *phoPQ*, *rpo*, *fur*	*avrA*, *csg*, *fim*, *pef*, *bcf*, *sth*, *lpf*, *inv*, *mgt*, *org*, *pip*, *prg*, sic, *sif*, *sip*, *sop*, *spa*, *spt*, *ssa*, *ssc*, *sse*, *slr*, *mig‐14*, *phoPQ*, *rpo*, *fur*	*avrA*, *csg*, *fim*, *pef*, *bcf*, *sth*, *lpf*, *inv*, *mgt*, *org*, *pip*, *prg*, sic, *sif*, *sip*, *sop*, *spa*, *spt*, *ssa*, *ssc*, *sse*, *slr*, *mig‐14*, *phoPQ*, *rpo*, *fur*	*avrA*, *csg*, *fim*, *pef*, *bcf*, *sth*, *lpf*, *inv*, *mgt*, *org*, *pip*, *prg*, sic, *sif*, *sip*, *sop*, *spa*, *spt*, *ssa*, *ssc*, *sse*, *slr*, *mig‐14*, *phoPQ*, *rpo*, *fur*
Origin and year of collection	Brazil, 2019	Brazil, 2019	Brazil, 2019	Brazil, 2019	Brazil, 2019	Brazil, 2019	Brazil, 2019	Brazil, 2019	Brazil, 2019	Brazil, 2019	Brazil, 2019	Brazil, 2019	Brazil, 2019	Brazil, 2019
GenBank/OneBr accession number	JABFEJ000000000	SAMN25026352	SAMN25026353	JABFEH000000000	SAMN25026350	SAMN25026351	JABFEG000000000	JABFEF000000000	SAMN25026354	SAMN25026355	SAMN25026356	SAMN25026357	SAMN25026358	SAMN25026359

*Note*: *Point mutation: ^1^p.S83F; ^2^p.T57S.

Abbreviation: SPIs, *Salmonella* pathogenicity island.

All isolates in a serovar featured similar genes conferring predicted resistance to multiple antimicrobials, with identical or more similar profiles observed when originated from the same farm (Table 1). The resistance gene profiles were the same for aminoglycosides, tetracyclines and sulfonamides (*n* = 14, 100%). The predicted in silico resistance to fluoroquinolones and beta‐lactamases detected these to be chromosomally or plasmid located (Tables S1 and S2). Predicted point mutations in genes encoding resistance to fluoroquinolones were found in all isolates, with mutation in the *gyrA* (S83F) and *parC* (T57S) detected in all SH, and mutations in *parC* (T57S) in all SM (Table 1).

Except for two SH, all isolates presented beta‐lactamase genes (*n* = 12/14, 85,7%), with AmpC beta‐lactamase (*bla*_CMY‐2_) being the predominant (*n* = 9/12, 75%) (Table 1). The *bla*_CTX‐M‐2_ type was detected in three SH isolates (Table 1).

SH harboured the fosfomycin‐resistant gene (*fosA7*) in all isolates (*n* = 10/10), while three SM isolates carried PMQR (*qnrB19*). Predicted resistance to disinfectants was represented with quaternary ammonium (*qacE*) and detected in SH (*n* = 3/14, 21.4%), and formaldehyde (*formA*) in SM (*n* = 1/14, 7%; Table 1). Screening of public genomes identified *formA* in 0.23% (*n* = 10/4395) among all SH and in 1% of all SM (*n* = 3/294), while for *qacE*, 14.8% (n = 649/4395) of SH were positive and 2% (*n* = 6/294) for SM (Tables S3 and S4).

Minimum inhibitory concentration (MIC) results confirmed the in silico detected resistance genes, with exceptions: no phenotypic fluoroquinolone resistance was observed in one isolate carrying only *parC* point mutation without PMQR, or for most of the isolates regarding aminoglycosides. All SH carrying the *fosA7* gene were susceptible to fosfomycin (MIC; Table S5).

### In silico plasmid characterization and conjugation assays

An array of plasmid types was detected in both groups of isolates (Table 1). The IncC plasmid type was present in all isolates (14/14), followed by ColRNAI (*n* = 8/14, 57%). The short‐read de novo plasmid analyses suggest that IncC plasmid replicons harboured beta‐lactamases (*bla*_CTX‐M‐2_, or *bla*_CMY‐2_) on isolates of both serovars (Tables S1 and S2), frequently hosting genes encoding resistance to other antibiotic classes (sulfonamides, tetracyclines, and aminoglycosides) (Figures S1 and S2). Conversely, only SM harboured PMQR, with the *qnrB19* gene likely located on ColRNAI plasmids without any other resistance genes (Figure S3).

The conjugation experiments with two SM isolates carrying AmpC beta‐lactamase (*bla*_CMY‐2_) and PMQR (*qnrB19*) confirmed the transfer of resistance to the recipient *E. coli* strain and to other genes predicted to be carried on the same plasmid (tetracyclines, sulfonamides). Plasmid profiling showed the expected plasmid sizes indicated by the de novo plasmid assembly. MIC confirmed the transfer of the plasmids and resistance levels for Beta‐lactams and fluoroquinolones (though lower for nalidixic acid), while also conferring resistance to sulphonamides and tetracyclines (Table 2).

**TABLE 2:** MIC levels according to the European Committee for Antibiotic Susceptibility (EUCAST, 2022) for the two donors of *Salmonella* Minnesota carrying AmpC beta‐lactamase and PMQR and the resulting *Escherichia coli* transconjugant strains

ID	Resistance genes of interest	Plasmid	MIC (mg/L)														
			AMP	CTX	CTZ	MER	NAL	CIP	GEN	AMI	TMP	SUL	COL	TET	TGC	CHL	AZI
Donor strain 319	*bla*CMY‐2, *qnrB19*,	IncC, ColRNAI	>32 (R)	>4 (R)	>8 (R)	=0.06 (S)	>32 (R)	>0.5 (R)	≤0.5 (S)	≤4 (S)	≤0.25 (S)	>512 (R)	=2 (S)	>32 (R)	=2 (R)	≤8 (S)	=4 (S)
Trasconjugant strain 319	*bla*CMY‐2, *qnrB19*,	IncC, ColRNAI	>32 (R)	>4 (R)	>8 (R)	≤0.03 (S)	≥16 (S)	>0.5 (R)	≤0.5 (S)	≤4 (S)	≤0.25 (S)	>512 (R)	=2 (S)	>32 (R)	=2 (R)	≤8 (S)	=4 (S)
Donor strain 320	*bla*CMY‐2, *qnrB19*,	IncC, ColRNAI	>32 (R)	>4 (R)	>8 (R)	=0.06 (S)	>32 (R)	>0.5 (R)	≤0.5 (S)	≤4 (S)	≤0.25 (S)	>512 (R)	=2 (S)	>32 (R)	=1 (R)	≤8 (S)	=8 (S)
Trasconjugant strain 320	*bla*CMY‐2, *qnrB19*,	IncC, ColRNAI	>32 (R)	>4 (R)	>8 (R)	≤0.03 (S)	≥ 16 (S)	>0.5 (R)	≤0.5 (S)	≤4 (S)	≤0.25 (S)	>512 (R)	≤1 (S)	>32 (R)	=1 (R)	≤8 (S)	=8 (S)
*Recipient strain E. coli* J53‐1	—	—	=1 (S)	≤0.25 (S)	≤0.25 (S)	≤0.03 (S)	≤4 (S)	≤0.015 (S)	≤0.5 (S)	≤4 (S)	=0.5 (S)	≤8 (S)	≤1 (S)	≤2 (S)	≤0.25 (S)	≤8 (S)	≤2 (S)

*Note*: The letter in parenthesis indicate susceptibility (S) or resistance (R).

Abbreviations: AMI, amikacin; AMP, ampicillin; AZI, azithromycin; CHL, chloramphenicol; CIP, ciprofloxacin; COL, colistin; CTX, cefotaxime; CTZ, ceftazidime; FOS, fosfomycin; GEN, gentamicin; MER, meropenem; MIC, minimum inhibitory concentration; NAL, nalidixic acid; SUL, sulfamethoxazole; TET, tetracycline; TGC, tigecycline; TMP, trimethoprim.

### Core genome phylogenies

**FIGURE 1:**
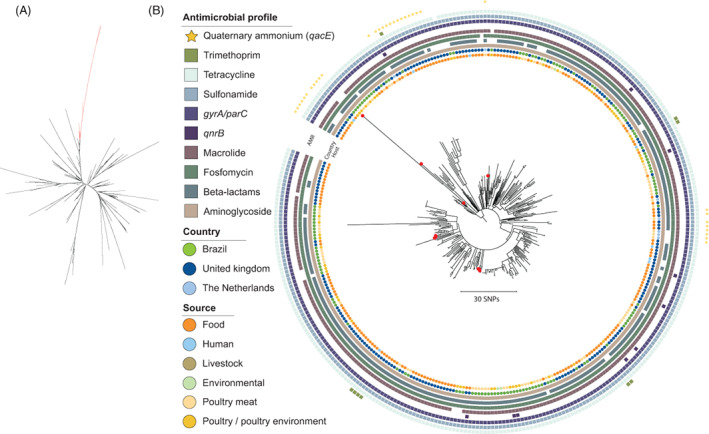
Phylogeny of *Salmonella* Heidelberg isolates based on core genome single nucleotide polymorphisms (SNPs) using *Salmonella* Heidelberg NC_011083 as a reference. (A) Unrooted tree based on 4385 worldwide genomes with branches in red highlighting the section where the study isolates and closely related clusters (<130 SNPs) are located. (B) Tree based on the highlighted section in a composed of 328 genomes and rooted at mid‐point. Classification according to in silico predicted antimicrobial profile, country, and source are coloured differently according to the figure's scheme from the outer to the inner rings. The study's sequences are marked with a red circle at the tip of the branch. The tree has been annotated and visualized using iTOL. Scalebar indicates the number of SNP differences

The study's Brazilian SH isolates where located in one distinct clade among international isolates in the phylogeny (Figure 1A). There was high variability between the 10 SH distributed across the phylogeny, with isolates sharing the same farm origin clustering in the same overall clades (Figure 1B). They clustered with other Brazilian poultry isolates (meat, poultry, and farm environment), isolates from the United Kingdom (unidentified food and human cases), and the Netherlands (poultry meat imported from Brazil).

SNP calling identified variant positions in 76.7% (3.96 Mb) of the reference chromosome. The lowest number of SNPs between the closest food isolate from the United Kingdom and an SH isolate of this study was 6 SNPs, whereas 31 SNPs were observed to a poultry meat isolate from the Netherlands. Between the closest human isolate (United Kingdom) and the study isolates, a minimum of 15 SNPs of difference were detected.

Overall, predicted AMR profiles among the entire SH phylogeny presented predicted resistance to aminoglycosides, fosfomycin, macrolides, tetracyclines, sulfonamides, and fluoroquinolones (point mutation), besides differences regarding beta‐lactamases and PMQR (Figure 1B).

**FIGURE 2:**
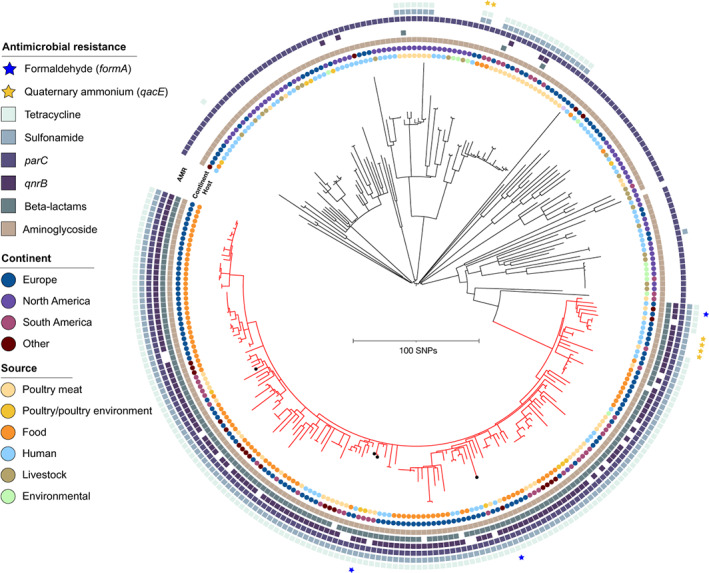
Phylogeny of *Salmonella* Minnesota isolates based on core genome single nucleotide polymorphisms. The tree is based on 248 genomes and rooted at mid‐point using *Salmonella* Minnesota NZ_CP017720.1 isolate as a reference. Classification according to in silico predicted antimicrobial profile, continent, and source are coloured differently according to the figure's scheme from the outer to the inner rings. The clade named Minnesota clonal expansion is highlighted in red. The study's sequences are marked with a circle at the tip of the branch. The tree has been annotated and visualized using iTOL. Scalebar indicates the number of single nucleotide polymorphisms (SNP) differences

**FIGURE 3:**
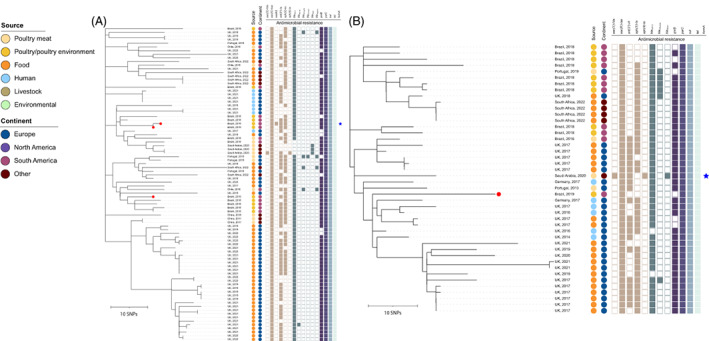
Phylogeny of *Salmonella* Minnesota isolates based on core genome single nucleotide polymorphisms (SNPs) highlighting the subclusters (A,B) where the isolates of this study are located in the Minnesota clonal expansion clade. The study's sequences are marked with a circle at the tip of the branch. The tree has been annotated and visualized using iTOL. Scalebar indicates the number of SNP differences

For SM, variant positions were identified in 81.4% (4.1 Mb) of the reference chromosome. Here, all isolates of this study did not vary significantly in their clustering according to different farm origins while also nesting in a single large clade, named MCE (Minnesota Clonal Expansion) separated by all other isolates by at least 140 SNPs. This clade was remarkably homogenous presenting short branches indicating the emergence of a successful clone, sharing very similar MDR profiles, and differing considerably from the topology of the remaining isolates in the phylogeny. MCE encompassed isolates of animal/food and human sources, different continents, and predicted resistance markers to fluoroquinolones (point mutations), and beta‐lactams (Figure 2). The four Brazilian isolates were located in two subclusters (≤75 SNPs in between) where they intermingled with isolates from poultry, food, and human cases. A connection with food isolates (United Kingdom and Portugal), and humans (United Kingdom) was observed (Figure 3A). The isolates from Portugal originated from imported poultry meat, likely from Brazil (Silveira et al., 2021). The other cluster depicted only one SM isolate from this study that nested among food isolates from the United Kingdom, Portugal (imported poultry meat), and human cases from the United Kingdom, and from Germany (Figure 3B). Clustering with food/poultry meat sources from other countries that import Brazilian poultry meat (South Africa and Saudi Arabia) was also observed in the MCE (Figures 3A,B).

The number of SNPs identified between the study's isolates and the closest food‐related isolates showed 21–42 SNPs to UK, and Portugal isolates. The closest human isolates (United Kingdom and Germany) varied between 24 and 45 SNPs. All isolates in MCE, shared similar AMR in silico profiles in terms of resistance to aminoglycosides, beta‐lactams (particularly *bla*_CMY‐2_), sulfonamides, fluoroquinolones, and tetracyclines (Figure 3A,B). Predicted resistance to formaldehyde was only observed in the MCE clade (Figure 2).

## DISCUSSION

In this study, we show the presence of isolates of SH and SM in faeces of asymptomatic broilers within the Brazilian poultry production that are MDR to high‐priority drugs. These isolates were closely related to poultry, food, and human isolates from other countries, particularly those that import Brazilian poultry meat.

Here, all isolates presented SPIs encoding virulence factors that may contribute to the development of invasive infections in humans. The expression of these genes allows the strains to survive the acidic environment of the gut; attach, invade, and replicate in epithelial cells and macrophages causing sepsis; secrete toxins and cause apoptosis; and evade the immune system (Wang et al., 2020). Monte et al. (2019) also identified several virulence factors here detected, in some SH and SM from Brmazilian poultry, but that study lacked some SPIs found in all our isolates, in particular SPI‐1 which plays an important part facilitating invasion and dissemination, though that absence does not indicate lesser capacity for virulence.

Among all NTS affecting humans, SH is the serovar most associated with high morbidity levels (EFSA Panel on Biological Hazards (EFSA BIOHAZ Panel), 2019). Despite the low numbers of reported human infections by SM, gastroenteritis cases do occur (European Centre for Disease Prevention and Control [ECDC], 2022; Silveira et al., 2021), as well as invasive conditions (Steinebrunner et al., 2013).

In this study, resistance to third‐generation beta‐lactams was widespread, predicted to be mainly located on IncC‐type plasmid replicons, and all isolates presented at least one point mutation linked to resistance to fluoroquinolones and/or PMQR (Table 1). In particular, extensive and similar resistance profiles were observed in the recently emerged MCE clade where all isolates were closely related (<75 SNPs) (Figure 2). A predominance of *bla*_CMY‐2_ associated with IncC plasmids both in SH and SM suggests this replicon to be the main cause of dissemination for AmpC beta‐lactamase whether nationally or in imported Brazilian poultry products. This is observed in all the main Brazilian poultry producing states, showing widespread flock colonization (Monte et al., 2019; van den Berg et al., 2019).

This study predicted in silico resistance to other main drug classes (aminoglycosides, tetracyclines, sulfonamides, and fosfomycin). Kipper et al., (2021) analysed SH from poultry and its environment in Brazil between 2014 and 2018, with similar resistance gene profiles to our study regarding tetracyclines (*tet*[A]), sulfonamides (*sul2*), fosfomycin (*fosA7*), aminoglycosides, and a predominance of *bla*_CMY‐2_. Similarly to our isolates, *sul2* and *tet*(A) were co‐located on IncC plasmids which frequently harboured *bla*_CMY‐2_ (Figure S2). IncC replicons were also predicted to contain *bla*_CTX‐M‐2_ in our SH, as well as co‐location of other resistance genes (Figure S1).

A genomic analysis of SH from imported Brazilian poultry meat in the Netherlands detected *bla*_CMY‐2_ in most isolates. All 122 isolates carried *parC* mutation and most (*n* = 118, 96.7%) also had *gyrA* mutation as our isolates also did. Most detected plasmid replicons were IncX1 and IncC, and only three isolates carried *qnrB19* (van den Berg et al., 2019). Like our isolates, *qnrB19* was also previously detected in SM from poultry meat in Brazil (Monte et al., 2019) and analyses in Portugal with imported poultry meat from Brazil both with SH and SM, showed similar results, with additional beta‐lactamase genes (Silveira et al., 2021).

Here, not all genes predicting MDR were expressed, particularly those coding for aminoglycosides and fosfomycin (Table S5). Though we detected several aminoglycoside resistance gene combinations, only two isolates expressed resistance (to gentamycin) which has also been reported among different *Salmonella* spp. (Neuert et al., 2018). SH from poultry carrying *fosA7* can express high resistance and experimentally transfer it to a recipient strain (Rehman et al., 2017). Phenotypic fosfomycin resistance prevalence in *Salmonella* spp. in Brazilian poultry is unknown, though *fosA* genes are increasingly detected (Kipper et al., 2021; Monte et al., 2019).

The same point‐mutations here detected in *gyrA* (p.S83F), *parC* (p.T57S), and *qnrB19*, have been reported in Brazilian poultry (de Melo et al., 2021; Monte et al., 2019) and all our isolates but one (which only harboured *parC*) showed complete or low‐level resistance to ciprofloxacine and resistance to nalidixic acid according to the breakpoints (European Committee on Antimicrobial Susceptibility Testing [EUCAST], 2022; Table S5). Different combinations of *gyrA*/*parC* with or without *qnrB19* and variable phenotypic resistance are described for *Salmonella* spp. (Neuert et al., 2018). Therefore, despite our isolates featuring MDR profiles, antibiotic alternatives for treating possible human clinical cases are still available.

Brazilian poultry meat contaminated with MDR SH and SM is a high‐risk route of introduction and establishment for these serovars into the EU (Campos et al., 2018; van den Berg et al., 2019). Germany, the Netherlands, and Portugal have been reporting an increase in *bla*_CMY‐2_, *bla*_CTX‐M_ types, and fluoroquinolone‐resistant SH and SM in imported poultry products from Brazil. These products enter the EU since the current regulative does not take into account NTS serovars other than *Salmonella* Typhimurium and *Salmonella* Enteritidis, and do not test for antimicrobial resistance carriage in additional NTS (Liakopoulos et al., 2016; Silveira et al., 2021). Brazil has some legislation on NTS, regarding monitoring to reduce prevalence of *Salmonella* spp. in poultry, growth‐promoters restriction and enhancing biosecurity (PAN‐BR, 2018‐2022). However, the use of several drug classes that are also used in human medicine, is allowed prophylactically and therapeutically, potentially contributing to co‐selection of MDR with serious consequences for the treatment of human infections (Rabello et al., 2020).

Our phylogenies likely support these findings linking to imported poultry meat/food, and also to human disease. Here, SH clustered with MDR isolates from animal sources (United Kingdom and The Netherlands) (Figure 1B), while SM also clustered with animal/food MDR isolates from the United Kingdom and Portugal (Figures 3A,B), besides the close relatedness of our isolates with human cases caused by SH (United Kingdom) and SM (United Kingdom and Germany; Figures 1B and 3A,B). Alikhan et al. (2022) reported that SH and SM are unlikely causing significant human disease in the United Kingdom, although MDR carriage on plasmids is of concern. Manges (2016) discussing Extraintestinal Pathogenic *E. coli* (ExPEC), mentioned that even if a small number of human infections is caused/related to MDR lineages from poultry, this should be of importance. As these plasmids are associated with MDR, enhancing the risks of resistance dissemination besides the virulent strains themselves, we assessed the conjugative potential of SM plasmids carrying *bla*_CMY‐2_, and *qnrB19*. Their transfer and confirmed phenotypes in the transconjugants (Table 2), illustrate the potential for resistance spreading. Conjugation assays with SH and SM (carrying *bla*_CMY‐2_) from imported poultry meat from Brazil have been demonstrated (Campos et al., 2018), and the transfer of *bla*_CMY‐2_ from poultry to human commensal bacteria has been shown to occur (Anjum et al., 2019).

This study analysed faecal samples, representing the avian gastrointestinal tract and the poultry environment without comparing isolates from meat products. However, it has been shown that both serovars have many adaptive characteristics, including response to the acidic environment and production of biofilms, persisting in the poultry organism and environment, contaminating the equipment and the final product, besides contaminating abattoirs effluents (Barros et al., 2007; Voss‐Rech et al., 2019). Genes involved in biofilm formation and acid tolerance were found in all of our isolates (Table 1). de Melo et al. (2021) analysed the in vitro biofilm formation in SM from Brazilian poultry detecting genes for adhesion (*agfA/csg*), biofilm formation (*lpfA*), and survival under oxidative stress (*sodC*). These were also present in our isolates, except for *sodC* in SM, exemplifying the variety of circulating strains, even if sampled in the same geographical regions (Southeast Brazil).

Resistance to quaternary ammonium compounds (*qacE*) and formaldehyde (*formA*) is still rarely reported in the poultry industry (Stefani et al., 2018). The gene *qacE* was detected in this study's SH (Table 1), and in public genomes from both serovars (Tables S3 and S4). Resistance to formaldehyde is less frequent in public genomes (Tables S3 and S4), but also present in one of our isolates (Table 1). Although formaldehyde in poultry production has up until now not been related to phenotypic resistance (Ricke et al., 2019), *formA* detection in public genomes deserves further attention. Disinfectant resistance in our isolates is present on the same plasmid contigs carrying resistance to several drug classes, and co‐selection could further contribute to spreading of MDR. Besides, *qac* genes are frequently found on plasmids associated to Class 1 integrons, facilitating co‐resistance transmission to multiple antibiotics (Buffet‐Bataillon et al., 2012). Long‐read sequencing in our isolates could better characterize the genomic context of these plasmids.

In summary, we report the antimicrobial resistance and similarity to international isolates of different sources of SH and SM isolates from Brazilian poultry. Their virulent and MDR profiles, associated with the presence of transmissible plasmids can represent serious risks for human health, also on an international level. The associated MDR and clonal dissemination regarding the MCE clade deserve additional attention. The findings were presented further point to a need for improved efforts to control these and other *Salmonella* serovars in Brazilian poultry.

## FUNDING INFORMATION

This research was funded by the Statens Serum Institut, Københavns Universitet, and Dansk regering stipendium under kulturelle aftaler (Andre Becker Simoes Saidenberg)‐Uddannelses og Forskningsministeriet. Additional funding by: Grand Challenges Explorations Brazil (OPP1193112), FAPESP (2020/08224‐9), and CNPQ (443819/2018‐1, 312249/2017‐9, and 433128/2018‐6), grants: 165385/2018‐9 (Leticia Soares Franco), 312249/2017‐9 (Nilton Lincopan), 306396/2020‐3 (Terezinha Knöbl). Doctoral fellowship (CAPES Finance Code 001) (Andre Becker Simoes Saidenberg).

## Supporting information


**Supplementary File S1** Experimental methods used to determine the phenotypic features, conjugation assays, and genomic‐based comparisons

**TABLE S1** Location for chromosomal or plasmidial resistance to beta‐lactamases and fluoroquinolones of *Salmonella* Heidelberg isolates and homology to GenBank sequences including country of isolation and host. The asterisk indicates point mutation resistance

**TABLE S2** Location for chromosomal or plasmidial resistance to beta‐lactamases and fluoroquinolones of *Salmonella* Minnesota isolates and homology to GenBank sequences including country of isolation and host. The asterisk indicates point mutation resistance

**TABLE S3** In silico predicted resistance for quaternary ammonium compounds (*qacE*) and formaldehyde (*formA*) for the *Salmonella* Heidelberg isolates analysed in this study and from public databases

**TABLE S4** In silico predicted resistance for quaternary ammonium compounds (*qacE*) and formaldehyde (*formA*) for the *Salmonella* Minnesota isolates analysed in this study and from public databases

**TABLE S5** Minimum inhibitory concentration levels according to the European Committee for Antibiotic Susceptibility (EUCAST 2022) for the isolates sampled in this study. The letter in parenthesis indicates susceptibility (S), or resistance (R)

**TABLE S6** Biosample ID and metadata from the NCBI/EnteroBase databases of *Salmonella* Heidelberg genome sequences used for phylogenetic comparisons

**TABLE S7** Biosample ID and metadata from the NCBI/EnteroBase databases of *Salmonella* Minnesota genome sequences used for phylogenetic comparisons

**FIGURE S1** BLAST atlas exemplifying the comparison using GView of plasmids carrying CTX‐M‐2 extended‐spectrum beta‐lactamase extracted from this study's *Salmonella* Heidelberg. The inner ring represents the plasmid reference (pESBL3227). The three outer rings aligned to the reference show *S*. Heidelberg isolates that carry *bla*_CTX‐M‐2_ and co‐resistance to aminoglycosides and sulfonamides

**FIGURE S2** BLAST atlas exemplifying the comparison using GView of plasmids carrying AmpC beta‐lactamase extracted from this study's *Salmonella* Minnesota (SM). The inner ring represents the plasmid reference (pEC009.1). The three outer rings aligned to the reference show SM isolates that carry *bla*_CMY‐2_, and co‐resistance to tetracyclines and sulfonamides

**FIGURE S3** BLAST atlas exemplifying the comparison using GView of plasmids carrying plasmid mediated quinolone resistance extracted from this study's *Salmonella* Minnesota (SM). The inner ring represents the plasmid reference (pQNR17). The three outer rings aligned to the reference show SM isolates that carry *qnrB19*

## Data Availability

The whole‐genome nucleotide sequence of the bacterial strains used in this work are available in the NCBI Sequence Read Archive (PRJNA715669), and OneBr database (http://onehealthbr.com): strains ONE415, ONE418, ONE419, and ONE420.
