# Supplementary material for: Salmonella Heidelberg and Salmonella Minnesota in Brazilian broilers: Genomic characterization of third‐generation cephalosporin and fluoroquinolone‐resistant strains
Source: Environ Microbiol Rep. 2023 Jan 11;15(2):119–28. doi: 10.1111/1758-2229.13132 (PMC10103857; doi:10.1111/1758-2229.13132)
Supplement: Supplementary file 1 — Supplementary File S1 Experimental methods used to determine the phenotypic features, conjugation assays, and genomic‐based comparisons [file EMI4-15-119-s003.docx]

**Supplementary file 1**

**Antimicrobial susceptibility and transconjugants plasmid profiling**

The minimum inhibitory concentration (MIC) for most antimicrobials, both for the 14 isolates and for the two isolates selected for the conjugation assay, was determined with the EUVSEC3 panel (Thermo Fisher Scientific, Massachusetts, USA). Fosfomycin MIC was determined using the agar dilution method with a commercial kit (Liofilchem, Teramo, Italy). Clinical breakpoints values followed the EUCAST guidelines (EUCAST, 2022). Transconjugant colonies were subjected to plasmid extraction using a Plasmid MiniPrep kit (Thermo Fisher Scientific), visualized in 0.8% (w/v) agarose gel and their sizes compared to reference plasmids carried by the *E. coli* control strains 39R861 (Threlfall et al., 1986) and V517 (Macrina et al., 1978).

**DNA library preparation, *de novo* genome assembly and *in silico* typing**

DNA extraction and purification of overnight cultures was done using a Maxwell RSC kit and automated instrument (Promega, Wisconsin, USA) following the manufacturer instructions. Libraries were created with a Nextera XT DNA Library kit (Illumina, San Diego, CA) generating paired-end 150 bp sequences in an Illumina MiSeq platform (Illumina) according to the manufacturer’s protocol. The reads of this study were uploaded in the NCBI Sequence Read Archive (PRJNA715669) or were previously uploaded at the OneBr database (http://onehealthbr.com/bacteria/SeBr), with individual accession numbers provided in Table 1.

Assembly was performed with Shovill (v.1.0.4, https://github.com/tseemann/shovill) using the SPAdes assembler (v.3.15.4). The assembled genomes were used for *in silico* typing utilizing settings with a minimum of 90% coverage and 90% identity. The virulence factors, serovar, plasmid replicons, and antimicrobial resistance genes, were identified using ABRicate v.0.9.0 (<https://github.com/tseemann/abricate>) and the databases VFDB, Plasmidfinder, and Resfinder databases from the Center for Genomic Epidemiology (<https://cge.cbs.dtu.dk/services>); NCBI’s Bacterial Antimicrobial Resistance Reference Gene Database (Feldgarden et al., 2019). Additional testing was done with the Virulence Factors of Pathogenic Bacteria database (<http://www.mgc.ac.cn/cgi-bin/VFs>), MLST (multi-locus sequence typing) with mlst v.2.16 (<https://github.com/tseemann/mlst>), and serotyping with SISTR v1.1.1 (Yoshida et al., 2019).

**Selection of representative genomes for the study**

To compare the sequenced isolates from our study with those available on public genome repositories (NCBI Reference Sequence Database (https://www.ncbi.nlm.nih.gov/refseq/) and EnteroBase (https://enterobase.warwick.ac.uk/) (accessed on 08/2022) the following approach was applied to obtain assembled draft genomes for inclusion: For *S.* Heidelberg, all available 4,385 genomes on EnteroBase from all sources were analyzed for genetic relatedness to the 10 *S.* Heidelberg genomes of this study employing core genome SNPs (as outlined below), and selecting isolates clustering in the part of the phylogeny that contained the Brazilian isolates (<130 SNPs). From this cluster, a total of 318 sequences possessing metadata on source and country of isolation were selected for a detailed comparison, see Supplementary table 6. For *S.* Minnesota, among all 398 available genomes at the databases, 294 sequences possessing complete metadata on host and country were selected for further comparisons (<320 SNPs among these isolates). These were chosen to represent livestock (mostly poultry), the environment, and all those from human sources, see Supplementary table 7.

**Phylogenetic analyses**

Analyses of the core-genome single nucleotide polymorphisms (SNPs) were performed with the NASP pipeline v.1.0.0 (Sahl et al., 2016), mapping against the chromosome reference sequences SL476 (GenBank accession ID NC_011083, *S.* Heidelberg) and CFSAN017963 (GenBank accession ID NZ_CP017720.1, *S.* Minnesota) in separate analyses. The references were selected based on well described genomes similar to this work’s isolates (a maximum of 130 SNPs for *S*. Heidelberg and 320 SNPs for *S.* Minnesota). SNPs were called with GATK v.4.2.2 and Gubbins v.2.1 removed the putative recombinant regions. To retain only high-quality SNPs, positions with <90% unambiguous variant calls and a depth of <10 in individual isolates with sequence data were excluded across the collection. IQ-TREE v.2.1.2 was applied to the SNP matrix constructing the phylogenetic trees using ModelFinder with 100 bootstraps. FigTree v.1.4.4 (http://tree.bio.ed.ac.uk/software/figtree/) and iTOL v.6.4 (https://itol.embl.de/) were used for visualization and adding metadata graphics.

**Plasmid reconstruction and *in silico* analyses**

PlasmidSpades was used to assemble the plasmids (Bankevich et al., 2012) on the raw reads and the resulting fasta files tested with PlasmidFinder (<https://cge.food.dtu.dk/services/PlasmidFinder/>) to detect the replicon types and ResFinder (<https://cge.cbs.dtu.dk/services/ResFinder/>) indicating the presence and location of the antimicrobial resistance genes with the standard settings. The plasmids contigs containing resistance genes to beta-lactams and fluoroquinolones, were compared for the presence in the same contigs for the genes encoding resistance to other antimicrobial classes. The contigs possessing the sequences for beta-lactams and fluoroquinolones were also extracted as fasta files and tested with QUAST (v.5.1) checking for the assembly quality as well as verifying the predicted *in silico* sizes for the specified assembled plasmid.

**Plasmid transferability**

Two *S.* Minnesota isolates from different integrations, positive for *bla*_CMY-2_ and *qnrB19*, besides possessing different overall AMR profiles (IDs 319 and 320, Table 1.) were chosen as donors for the conjugation experiments, testing for transferability of resistance to third generation cephalosporins and PMQR with the plasmid-free *Escherichia coli* recipient strain (J53-1 resistant to rifampicin). A methodology adapted from Khajanchi et al. (2019) was used to evaluate the transfer of plasmids. Briefly, the donor and the recipient were cultivated separately overnight in LB (Luria Bertani) broth with shaking. These were mixed in a 1:5 ratio (donor:recipient) according to their optical density. The mixture was incubated overnight and then 100 μl added to each selection plates (agar containing 50 μg/mL ampicillin+50 μg/mL rifampicin, or 0.5μg/mL ciprofloxacin and 50 μg/mL rifampicin) and incubated 24 hours. The resulting conjugation mix was suspended in 1mL LB broth and serial diluted for plate counting on the selective agar plates containing 50 μg/mL ampicillin or 0.5 μg/mL ciprofloxacin, and on 50 μg/mL ampicillin + 50 μg/mL rifampicin, or 0,5 μg/mL ciprofloxacin + 50 μg/mL rifampicin.

**References**

Bankevich, A., Nurk, S., Antipov, D., Gurevich, A.A., Dvorkin M, Kulikov, A.S., *et al*. (2012) SPAdes: a new genome assembly algorithm and its applications to single-cell sequencing. J Comput Biol 19: 455-477.

EUCAST. European Committee on Antimicrobial Susceptibility Testing. (2022) Clinical breakpoints - breakpoints and guidance**.** [WWW document]. URL www.eucast.org/clinical_breakpoints

Khajanchi, B.K., Kaldhone, P.R., and Foley, S.L. (2019). Protocols of conjugative plasmid transfer in *Salmonella*: plate, broth, and filter mating approaches. Methods Mol Biol 2016: 129-139.

Macrina, F. L., Kopecko, D. J., Jones, K. R., Ayers, D. J., and McCowen, S. M. (1978). A multiple plasmid-containing *Escherichia coli* strain: convenient source of size reference plasmid molecules. *Plasmid* 1, 417–420.

Sahl, J.W., Lemmer, D., Travis, J., Schupp, J.M., Gillece, J.D., Aziz, M., *et al*. (2016) NASP: an accurate, rapid method for the identification of SNPs in WGS datasets that supports flexible input and output formats. Microb Genom 2: e000074.

Threlfall, E. J., Rowe, B., Ferguson, J. L., and Ward, L. R. (1986). Characterization of plasmids conferring resistance to gentamicin and apramycin in strains of *Salmonella typhimurium* phage type 204c isolated in Britain. *J. Hyg.* 97, 419–426.

Yoshida, C., Kruczkiewicz, P., Laing, C.R., Lingohr, E.J., Gannon, V.P.J., Nash, J. H.E., *et al*. (2016) The *Salmonella In silico* typing resource (SISTR): an open web-accessible tool for rapidly typing and subtyping draft *Salmonella* genome assemblies. PLoS ONE 11: e0147101.
