# Supplementary material for: Salmonella Heidelberg and Salmonella Minnesota in Brazilian broilers: Genomic characterization of third‐generation cephalosporin and fluoroquinolone‐resistant strains
Source: Environ Microbiol Rep. 2023 Jan 11;15(2):119–28. doi: 10.1111/1758-2229.13132 (PMC10103857; doi:10.1111/1758-2229.13132)
Supplement: Supplementary file 3 — TABLE S2 Location for chromosomal or plasmidial resistance to beta‐lactamases and fluoroquinolones of Salmonella Minnesota isolates and homology to GenBank sequences including country of isolation and host. The asterisk indicates point mutation resistance [file EMI4-15-119-s006.docx]

| **Strain ID** | **309** | **318** | **319** | **320** |
| --- | --- | --- | --- | --- |
| **Beta-lactamase** | IncC plasmid *(bla*_CMY-2_*)* | IncC plasmid *(bla*_CMY-2_*)* | IncC plasmid *(bla*_CMY-2_*)* | IncC plasmid *(bla*_CMY-2_*)* |
| **Fluoroquinolone** | chromosome *(parC*)* | chromosome *(parC*) /* ColRNAI plasmid *(QnrB19)* | chromosome *(parC*) /* plasmid *(QnrB19)* | chromosome *(parC*) /* ColRNAI plasmid *(QnrB19)* |
| **Plasmid homology** | CP049350.1 | CP049350.1 / CP081650.1 | CP049350.1 / CP081650.1 | CP049350.1 / CP081650.1 |
| **Country of described plasmid** | China | China / Germany | China / Germany | China / Germany |
| **Host of described plasmid** | *Gallus gallus* | *Gallus gallus / Sus scrofa* | *Gallus gallus / Sus scrofa* | *Gallus gallus / Sus scrofa* |
