# Supplementary material for: Salmonella Heidelberg and Salmonella Minnesota in Brazilian broilers: Genomic characterization of third‐generation cephalosporin and fluoroquinolone‐resistant strains
Source: Environ Microbiol Rep. 2023 Jan 11;15(2):119–28. doi: 10.1111/1758-2229.13132 (PMC10103857; doi:10.1111/1758-2229.13132)
Supplement: Supplementary file 6 — TABLE S5 Minimum inhibitory concentration levels according to the European Committee for Antibiotic Susceptibility (EUCAST 2022) for the isolates sampled in this study. The letter in parenthesis indicates susceptibility (S), or resistance (R) [file EMI4-15-119-s010.docx]

| **ID** | **AMI** | **AMP** | **AZI** | **CTX** | **CTZ** | **CHL** | **CIP** | **COL** | **GEN** | **MER** | **NAL** | **SUL** | **TET** | **TIG** | **TMP** | **FOS** |
| --- | --- | --- | --- | --- | --- | --- | --- | --- | --- | --- | --- | --- | --- | --- | --- | --- |
| **289** | <=4(S) | >32(R) | =8(S) | >4(R) | >8(R) | <=8(S) | =0.25* | <=1(S) | <=0.5(S) | <=0.03(S) | >32(R) | >512(R) | >32(R) | =0.5(S) | <=0.25(S) | =0.25(S) |
| **292** | <=4(S) | >32(R) | =8(S) | >4(R) | >8(R) | <=8(S) | >0.5(R) | <=1(S) | <=0.5(S) | <=0.03(S) | >32(R) | >512(R) | >32(R) | =0.5(S) | <=0.25(S) | =1(S) |
| **293** | <=4(S) | >32(R) | =8(S) | >4(R) | >8(R) | <=8(S) | =0.25* | <=1(S) | <=0.5(S) | <=0.03(S) | >32(R) | >512(R) | >32(R) | =0.5(S) | <=0.25(S) | =0.25(S) |
| **294** | <=4(S) | >32(R) | =8(S) | >4(R) | >8(R) | <=8(S) | =0.25* | <=1(S) | <=0.5(S) | =0.06(S) | >32(R) | >512(R) | >32(R) | =0.5(S) | <=0.25(S) | =0.5(S) |
| **297** | <=4(S) | >32(R) | =8(S) | >4(R) | >8(R) | <=8(S) | =0.25* | <=1(S) | >16(R) | <=0.03(S) | >32(R) | >512(R) | >32(R) | =0.5(S) | <=0.25(S) | =1(S) |
| **300** | <=4(S) | >32(R) | =8(S) | >4(R) | >8(R) | <=8(S) | >0.5(R) | <=1(S) | >16(R) | <=0.03(S) | >32(R) | >512(R) | >32(R) | <=0.25(S) | =0.5(S) | =1(S) |
| **304** | <=4(S) | >32(R) | =8(S) | >4(R) | >8(R) | <=8(S) | >0.5(R) | <=1(S) | <=0.5(S) | <=0.03(S) | >32(R) | >512(R) | >32(R) | =1(R) | <=0.25(S) | =1(S) |
| **305** | <=4(S) | >32(R) | =8(S) | >4(R) | >8(R) | <=8(S) | =0.25* | <=1(S) | <=0.5(S) | =0.06(S) | >32(R) | >512(R) | >32(R) | =0.5(S) | <=0.25(S) | =2(S) |
| **306** | <=4(S) | =2(S) | =4(S) | <=0.25(S) | <=0.25(S) | <=8(S) | =0.25* | <=1(S) | <=0.5(S) | <=0.03(S) | >32(R) | >512(R) | >32(R) | =0.5(S) | <=0.25(S) | =1(S) |
| **307** | <=4(S) | <=1(S) | =8(S) | <=0.25 | <=0.25(S) | <=8(S) | >0.5(R) | <=1(S) | <=0.5(S) | =0.06(S) | >32(R) | >512(R) | >32(R) | =0.5(S) | <=0.25(S) | =1(S) |
| **309** | <=4(S) | >32(R) | <=2(S) | >4(R) | >8(R) | <=8(S) | <=0.015(S) | <=1(S) | <=0.5(S) | <=0.03(S) | <=4(S) | >512(R) | >32(R) | =0.5(S) | <=0.25(S) | =0.25(S) |
| **318** | <=4(S) | >32(R) | <=2(S) | >4(R) | >8(R) | <=8(S) | =0.25(S) | <=1(S) | <=0.5(S) | <=0.03(S) | >32(R) | >512(R) | >32(R) | =0.5(S) | <=0.25(S) | =0.25(S) |
| **319** | <=4(S) | >32(R) | =4(S) | >4(R) | >8(R) | <=8(S) | >0.5(R) | =2(S) | <=0.5(S) | =0.06(S) | >32(R) | >512(R) | >32(R) | =2(R) | <=0.25(S) | =0.25(S) |
| **320** | <=4(S) | >32(R) | =8(S) | >4(R) | >8(R) | <=8(S) | >0.5(R) | =2(S) | <=0.5(S) | =0.06(S) | >32(R) | >512(R) | >32(R) | =1(R) | <=0.25(S) | =0.25(S) |
| **ATCC_25922** | <=4(S) | =4(S) | =4(S) | <=0.25(S) | <=0.25(S) | <=8(S) | <=0.015(S) | <=1(S) | <=0.5(S) | <=0.03(S) | <=4(S) | =32(S) | <=2(S) | <=0.25(S) | =1(S) | =0.5(S) |

*The isolates marked with an asterisk indicate low-level ciprofloxacin resistance (>0.06g mg/L).

Legend: AMI (amikacin), AMP (ampicillin), AZI (azithromycin), CTX (cefotaxime), CTZ (ceftazidime), CHL (chloramphenicol), CIP (ciprofloxacin), COL (colistin), GEN (gentamicin), MER (meropenem), NAL (nalidixic acid), SUL (sulfamethoxazole), TET (tetracycline), TGC (tigecycline), TMP (trimethoprim), FOS (fosfomycin)
