# Supplementary figures and images for: Salmonella Heidelberg and Salmonella Minnesota in Brazilian broilers: Genomic characterization of third‐generation cephalosporin and fluoroquinolone‐resistant strains
Source: Environ Microbiol Rep. 2023 Jan 11;15(2):119–28. doi: 10.1111/1758-2229.13132 (PMC10103857; doi:10.1111/1758-2229.13132)

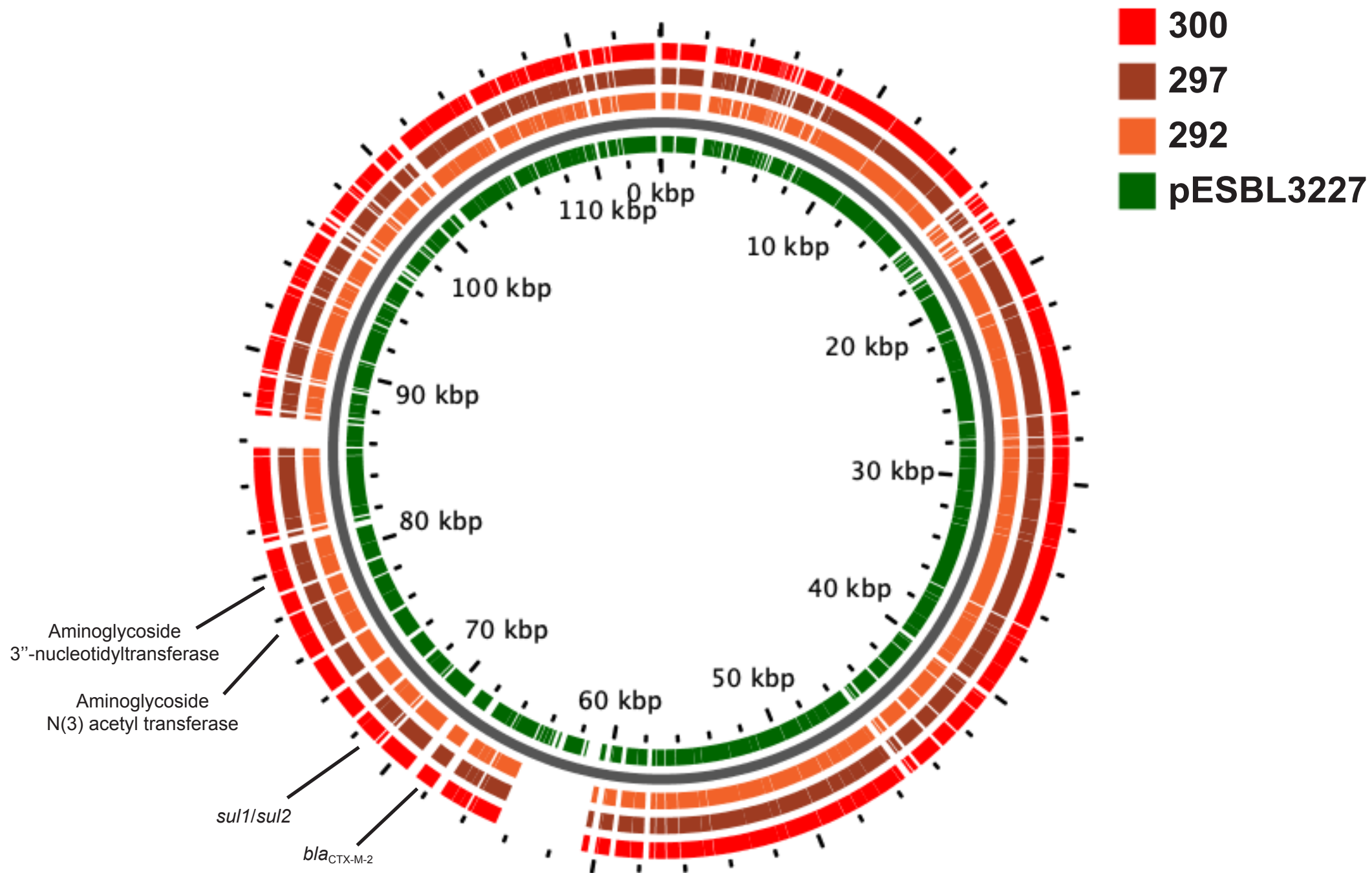

Supplement: Supplementary file 9 — FIGURE S1 BLAST atlas exemplifying the comparison using GView of plasmids carrying CTX‐M‐2 extended‐spectrum beta‐lactamase extracted from this study's Salmonella Heidelberg. The inner ring represents the plasmid reference (pESBL3227). The three outer rings aligned to the reference show S. Heidelberg isolates that carry bla CTX‐M‐2 and co‐resistance to aminoglycosides and sulfonamides [file EMI4-15-119-s002.pdf]

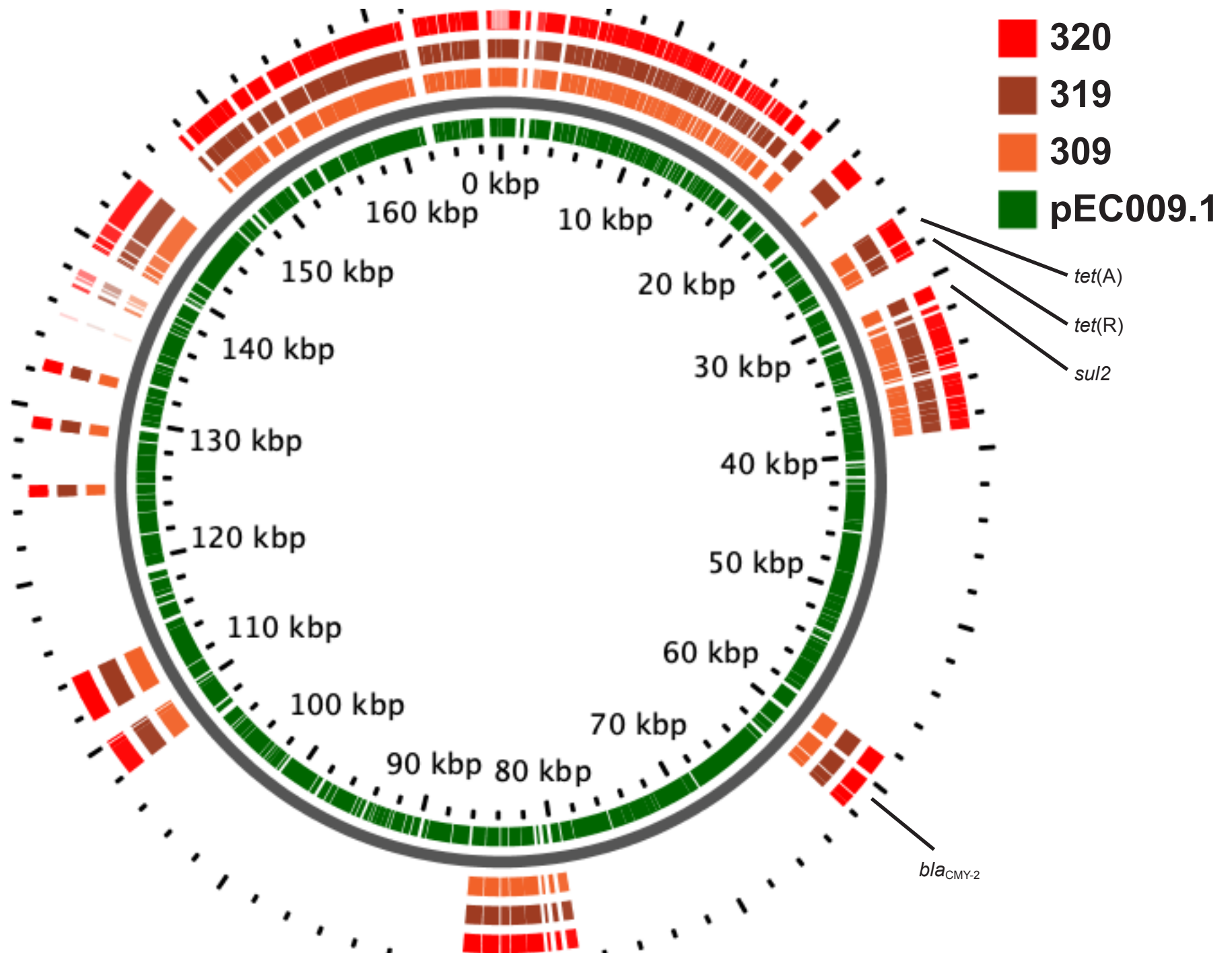

Supplement: Supplementary file 10 — FIGURE S2 BLAST atlas exemplifying the comparison using GView of plasmids carrying AmpC beta‐lactamase extracted from this study's Salmonella Minnesota (SM). The inner ring represents the plasmid reference (pEC009.1). The three outer rings aligned to the reference show SM isolates that carry bla CMY‐2, and co‐resistance to tetracyclines and sulfonamides [file EMI4-15-119-s009.pdf]

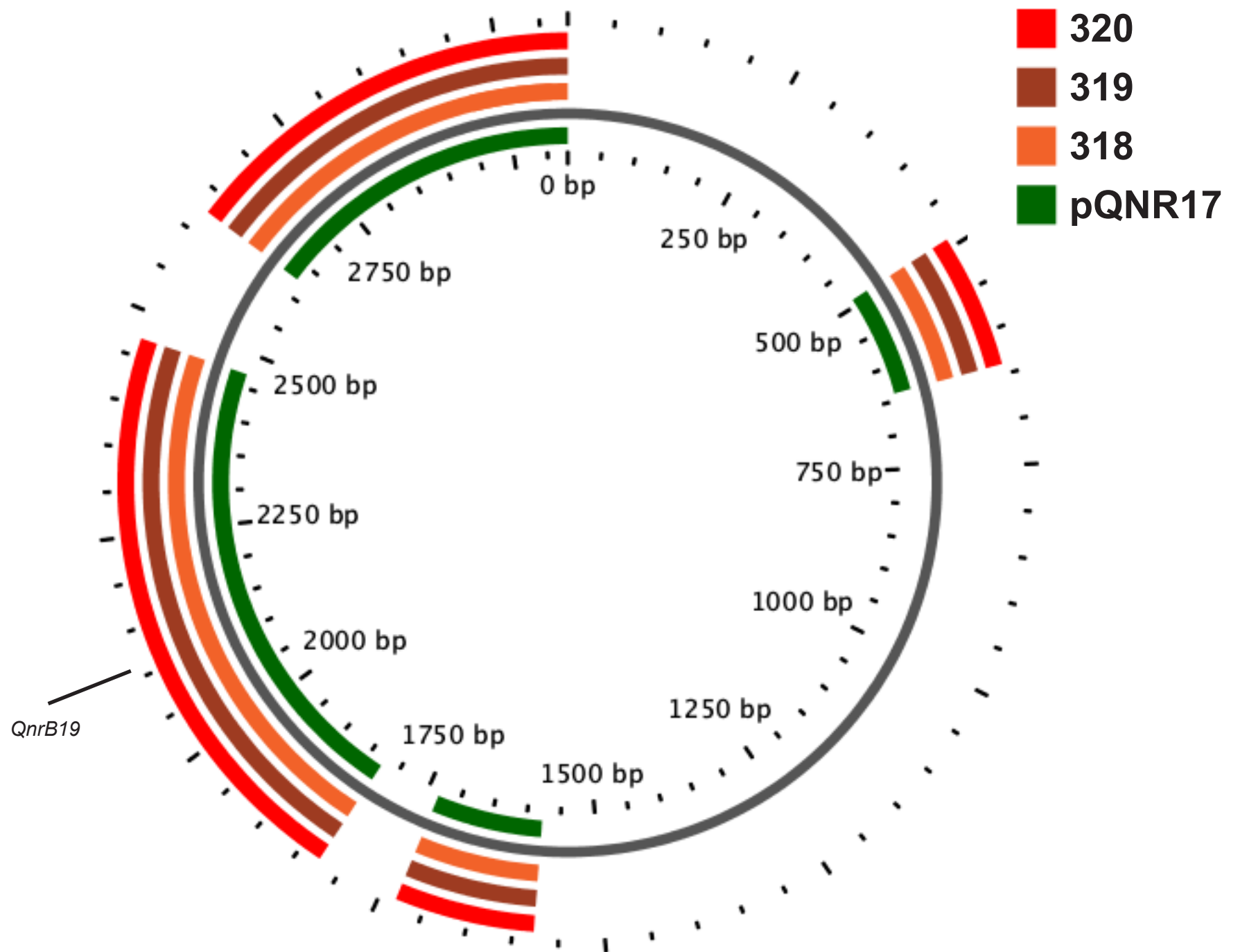

Supplement: Supplementary file 11 — FIGURE S3 BLAST atlas exemplifying the comparison using GView of plasmids carrying plasmid mediated quinolone resistance extracted from this study's Salmonella Minnesota (SM). The inner ring represents the plasmid reference (pQNR17). The three outer rings aligned to the reference show SM isolates that carry qnrB19 [file EMI4-15-119-s001.pdf]
